# Psychiatry Residents in China: Socio-Demographic Characteristics, Career Satisfaction, and Related Factors

**DOI:** 10.3389/fpsyt.2019.00177

**Published:** 2019-04-02

**Authors:** Feng Jiang, Huixuan Zhou, Linlin Hu, Jeffrey Rakofsky, Tingfang Liu, Shichao Wu, Huanzhong Liu, Yuanli Liu, Yi-lang Tang

**Affiliations:** ^1^Public Health School, Peking Union Medical College, Chinese Academy of Medical Sciences, Beijing, China; ^2^Department of Psychiatry and Behavioral Sciences, Emory University, Atlanta, GA, United States; ^3^Institute for Hospital Management of Tsinghua University, Beijing, China; ^4^Department of Psychiatry, Chaohu Hospital of Anhui Medical University, Hefei, China; ^5^Atlanta VA Medical Center, Decatur, GA, United States

**Keywords:** China, psychiatry residents, socio-demographic characteristics, satisfaction, workforce, intention to quit

## Abstract

**Objective:** To study the socio-demographic characteristics, the working environment, and the level of career satisfaction among psychiatry residents in China.

**Method:** This was a part of a large-scale, nation-wide online survey of hospitals, healthcare professionals, and patients. Data, including socio-demographics, work hours, income, job satisfaction, and intention to quit were collected anonymously. Respondents also completed the Minnesota Satisfaction Questionnaire (MSQ).

**Results:** One thousand sixty residents nested in 32 psychiatric hospitals in 29 provinces in China completed the survey. Respondents were predominantly female (69.0%), worked an average of 47.8 ± 11.6 h per week, and 28.97% had experienced medical disputes in the previous year. The top three reported reasons for dissatisfaction were low pay (50.1%), contentious doctor-patient relationships (17.6%), and high workload (10.8%). An intention to quit their current job was reported by 18.7% of residents. The overall mean MSQ score was 73.8 ± 13.8, with significant differences across post-graduate training years and regions. A multilevel regression analysis found that a higher MSQ score was significantly associated with fewer years in residency, shorter work hours, higher monthly pay, having medical liability insurance, and feeling satisfied with the level of doctor-nurse cooperation, their hospitals' medical disputes prevention/management, and the healthcare workers' social environment.

**Conclusion:** Psychiatry residents in China are predominantly female and well-educated. They are only moderately satisfied with their career. Poor salaries, contentious doctor-patient relationships and high workload are among their top complaints and may explain why a considerable proportion are intending to leave their current residency. More support from the government regarding residents' salaries, workload and working environment may help improve their job satisfaction and retention, ensuring that China will have a pool of well-trained and engaged psychiatrists for the future.

## Introduction

Despite a progress in recent years, the number of psychiatrists in China is still very limited and unevenly distributed throughout the country (1). One survey showed that by the end of 2010, there were ~20,480 psychiatrists which corresponds to 1.46 psychiatrists per 100,000 people (1). Compared to other countries, these numbers were much lower (2). Therefore, it is important to attract medical students to the field and learn more about those who have decided to pursue psychiatry residency training.

As is true in the United States and elsewhere, residency training in China can be stressful (3–6). A demanding workload, long work hours and other factors can cause burn-out and even dropout from residency programs (7). On the other hand, job satisfaction in residents, besides affecting the retention rate, may also be associated with increased resident effectiveness and lower absenteeism (7).

A literature review on the subject shows that there have been no nation-wide, large-scale studies of the socio-demographic characteristics and level of job satisfaction of psychiatry residents' training in China. A few surveys of psychiatrists or mental health professionals have been published but do not address psychiatry residents specifically. A few studies of non-psychiatry residents in China have also been published. For example, one survey of 135 internal medicine residents in Shanghai, China (8) found the turnover intention was relatively high (45.9%) and was predicted by the amount of job burnout. Another study in Fujian province (9) found that common reasons for job dissatisfaction reported by residents in internal medicine and other specialties were lack of support from friends and families, high expectations from the public and poor salaries.

Given the lack of data characterizing psychiatry residents training in China and the impairing nature of burnout, we conducted a nation-wide survey to determine the socio-demographic characteristics, the working environment, and the amount of career satisfaction among psychiatry residents training in China.

## Methods

### Study Design

This study was an anonymous survey conducted online from December 18th to December 29th, 2017. WeChat, a popular social media platform in China, was used to distribute the questionnaire and collect the data. The questionnaire was answered individually through participants' cell phones or computers. All target hospitals where the residents trained were contacted individually and the survey background, significance, instructions, and survey pathway were provided to ensure a high participation rate. The study was reviewed and approved by the Ethics Committee (IRB) at the Public Health School of Peking Union Medical College. Informed consent was provided on the homepage of the online survey and was obtained before participants completed the online survey. To avoid repeated response, an IP address could only be used once to access and complete the survey.

### Participants

This study was part of a larger research project, the National Hospital Performance Evaluation Survey, which was sponsored by the Public Health School of Peking Union Medical College and aimed to improve healthcare quality and satisfaction within China's psychiatric hospitals. The target hospitals were selected by the National Hospital Performance Evaluation Survey, and all respondents were targeted and reached by each hospital's administration team.

Psychiatry residents' training at the largest provincial psychiatric hospital in each capital city within the province were surveyed, except in Beijing where psychiatry residents from 3 hospitals were selected and in the Anhui Province, where those from 2 hospitals were selected. In total, 32 psychiatric hospitals from 29 provinces were selected (10). Psychiatric hospitals in the capital cities of Gansu province and Tibet were not included as none existed at the time of this study. According to the latest guidelines of the National Bureau of Statistics of China, the 29 provinces were divided into 4 regions: East China, Central China, West China and Northeast China (11).

### Questionnaire Design

The questionnaire was first developed by three authors (FJ, YLT, and HZL) independently of the surveyed hospitals, government bodies, or industry groups and based on a literature review. Then, a pilot study was conducted to gather users' feedback on the questions and wording to improve the questionnaire's readability and reliability. The final version was composed of a total of 51 questions. To ensure confidentiality, participants responded anonymously and requests for personal identifiable information were kept to a minimum. Open-ended questions were also included, such as the factors contributing to job satisfaction or dissatisfaction. The questionnaire was divided into three sections: basic personal and professional characteristics; (2) work environment; (3) job satisfaction and quality of life.

We assessed job satisfaction using the short version of the Minnesota Satisfaction Questionnaire (MSQ) (12), which measures general job satisfaction along two dimensions: intrinsic and extrinsic satisfaction. The scale has 20 items with Likert-type scaling, ranging from 1 (very dissatisfied) to 5 (very satisfied). The Chinese version has been widely used and has demonstrated good reliability and validity (13, 14).

### Statistical Analysis

After the online survey was closed, all data were exported to the IBM Statistical Package of Social Sciences (SPSS, version 22.0) for data management and statistical analysis. Summary statistics, including percentages and measures of central tendency, were used to describe the data. Statistical inference testing was performed using the Mann-Whitney *U*-test and Kruskal-Wallis test for the non-normal continuous variables and analysis of variance (15) and *t*-test were used for the normal continuous variables. Chi-square tests were used for unordered categorical variables. For each item reported, regional, and gender differences were assessed. When comparing residents' income across the different regions of China, the incomes were standardized to account for variability using the 2016 local city average incomes based on the published data (16), and Beijing's income was used as a benchmark. We also calculated an income index to measure the income level in the local city, which was a ratio of the income divided by local city average income.

Due to the fact that psychiatry residents were nested in 32 hospitals, and that the null model indicated that the intra-class correlation (17) was 14%, a multilevel modeling analysis was necessary to explore the associated factors with job satisfaction (18). This study applied two-level multivariate linear regression models, which allowed the authors to explore correlations within the hospitals (19), with individual as the low level factor and hospital as the high level factor. The models were run in SPSS for restricted maximum likelihood estimation and the −2 Log Likelihood was used for significance testing. A smaller number indicates a better model. In the analytical process, we added independent variables sequentially. First, we used the model with only the random intercept to assess whether there was significant variation in job satisfaction across hospitals and to reveal its size (Model 1). Then, in Model 2, we added individual-level independent variables. Finally, in Model 3, we added hospital-level variables. Statistical significance was set at 0.05 and all tests were two-tailed.

## Results

In total, there were 1,508 psychiatry residents in the selected hospitals and they were all invited to participate. 1,106 responded to the questionnaires, with a response rate of 73.3% and 1,060 completed the questionnaire in its entirety.

### Socio-Demographic Characteristics of Psychiatry Residents in China

More than two thirds (69.0%) of respondents were female and more than half (56.1%) were between 25 and 29 years old (range = 22–34 years old, mean 28.3 ± 2.6 years old).

Less than half (47.2%) were married and about one quarter (25.1%) had children. The largest proportion of participants (2/5) were from the heavily populated area of East China.

With regard to education, more than one third (34.3%) had post-graduate degrees in addition to their medical degrees, including additional master's degrees (31.0%) and Ph.D. degrees (3.3%) (see Table 1).

**Table 1:** Demographic characteristics of psychiatry residents (*N* = 1,060).

**Demographic variables**	**Frequency**	**%**
**GENDER**		
Female	731	69.0
Male	329	31.0
**AGE RANGE (YEARS)**		
≤24	95	9.0
25~29	595	56.1
≥30	370	34.9
**GRADE**		
PGY-1	316	29.8
PGY-2	214	20.2
PGY-3	214	20.2
PGY-4	114	10.8
PGY-5	202	19.1
**MARITAL STATUS**		
Married	500	47.2
Single	560	52.8
**CHILDREN**		
None	794	74.9
One or more	266	25.1
**EDUCATIONAL LEVEL**		
College/medical school	696	65.7
Master's Degree	329	31.0
MD or PhD Degree	35	3.3
**REGION**		
East China	435	41.0
Central China	224	21.1
West China	319	30.1
Northeast China	82	7.7

### Working Hours and Income

The average working hours per week were 47.8 ± 11.6, and there was no significant difference between male and female residents. Significant regional differences were found with residents in West China working significantly longer hours (50.2 ± 13.8), compared with colleagues in other regions (East China, 46.6 ± 9.6; Central China, 48.2 ± 12.2; Northeast China, 43.3 ± 6.8, *p* < 0.001).

In this survey, 57.7% of residents worked more than 40 h per week. Again, significant regional differences existed, with a significantly higher percentage of participants in West China (66.5%) working more than 40 h/week (East China, 57.7%; Central China, 54.9%; Northeast China, 31.7%, *p* < 0.001).

Both the actual and expected monthly incomes varied widely in the sample. The median monthly income of the participants was 8,518.9 RMBs. Those who worked in Central China reported a significantly higher income (median = 9501.0 RMBs) compared with those working in other regions while those in Northeast China reported the lowest (median = 7553.1 RMBs, *p* < 0.001). With respect to gender, we found no significant difference in actual and expected income level between male and female residents (see Table 2). Fewer than one third (30.8%) of residents reported income at or above the local average income level. The income index ranged from 0.05 to 2.13, with a mean of 0.86.

**Table 2:** Monthly standardized pay and expected standardized pay of psychiatrists (*N* = 1,060).

**Variables**	**Median**	**P25**	**P75**	***p***
**MONTHLY STANDARDIZED PAYMENT IN PREVIOUS YEAR (RMB)**				
Total	8518.9	6822.9	10891.4	
Male	8378.3	6961.0	10889.0	0.765^a^
Female	8528.6	6805.0	10918.2	
East China	8165.9	6375.6	10000.0	<0.001^b^^***^
Central China	9501.50	7675.76	11940.07	
West China	8816.8	6821.9	10929.5	
Northeast China	7553.1	5884.1	8794.2	
PGY-1	6961.0	5371.2	8509.1	
PGY-2	8938.9	7050.6	10921.0	
PGY-3	9107.9	7207.4	11523.8	<0.001^b^^***^
PGY-4	9267.3	7915.5	11830.7	
PGY-5	10120.2	8161.7	11865.2	
**EXPECTED STANDARDIZED MONTHLY PAYMENT (RMB)**				
Total	16532.6	12740.1	29628.5	
Male	17275.5	13777.2	21836.4	0.03^a^^*^
Female	16331.9	12343.5	20414.8	
East China	15279.6	12000.0	20372.8	<0.001^b^^***^
Central China	17275.5	14978.3	20730.5	
West China	17402.6	12751.1	24120.1	
Northeast China	14560.1	10523.3	18200.1	
PGY-1	13836.2	10186.4	20000.0	
PGY-2	16135.4	12129.4	20063.9	
PGY-3	17275.5	13777.2	20730.5	<0.001^b^^***^
PGY-4	17936.8	14560.1	24745.1	
PGY-5	18200.1	14880.0	25960.9	

a *Mann-Whitney U-test*.

b *Kruskal-Wallis test. UDS/RMB ratio 6.5*.

* *p <0.05*.

*** *p <0.001*.

Monthly standardized pay = monthly pay/2016 local city average monthly income*2016 Beijing city average monthly income.

Expected monthly standardized pay = expected monthly pay/2016 local city average monthly income*2016 Beijing city average monthly income.

*2016 local city average income data source: National Bureau of Statistics of China. http://data.stats.gov.cn/easyquery.htm?cn=E0105*.

### Experiences of Medical Disputes and Medical Liability Insurance

In this survey, medical disputes included all incidents involving complaints or grievances reported by patients or their families regarding medical services, treatment outcome or medical expenses, which may or may not actually represent medical malpractice (10).

Overall, 28.9% of residents experienced medical disputes and complaints in the previous year, with 33.4% of male residents and 26.8% of female residents reporting these experiences (*P* < 0.05). Central China had the greatest percentage of psychiatry residents (35.3%) experiencing these disputes, followed by West China (29.5%), East China (26.4%), and Northeast China (22.0%). These differences were not significant.

52.7% were satisfied with how the hospitals handled disputes or complaints made against the residents. Significant differences in satisfaction with this process were observed among the various regions with Northeast China having the highest rate of satisfied residents (69.5%), followed by Central China (53.6%), East China (53.6%), and West China (46.7%, *p* = 0.003). There were no differences between the genders.

The mean number of residents with medical liability insurance coverage was slightly more than half of participants (59.4%) and the rate varied widely from one region to another, ranging from 53.3% (West China) to 72.0% (Northeast China), *p* < 0.001.

### Perception of Support and Respect From the Society

77.3% of psychiatry residents were dissatisfied with the social environment (social acceptance, perceived respect and stigma) for healthcare workers, with Central China having a significantly higher percentage (81.7%) of dissatisfied residents and Northeast China having the lowest percentage (62.2%, *p* < 0.05). No gender differences were found.

65.0% did not believe that they received their due respect from the general public, with Central China having the highest percentage endorsing this belief (68.8%), followed by West China (67.7%), East China (63.7%), and Northeast China (51.2%, *p* < 0.05). No significant gender differences were found.

77.6% were satisfied with the quality of teamwork between doctors and nurses (regional rates: East China, 83.2%; Central China, 74.6%; West China, 69.9%; Northeast China, 86.6%, respectively, *p* < 0.001), and 77.0% were satisfied with the level of trust between doctors and nurses (regional rates: East China, 82.5%; Central China, 75.0%; West China, 68.0%; Northeast China, 78.8%, respectively, *p* < 0.001). No gender differences were found.

### Self-Reported Quality of Life and Health Status

Seventeen percentage of psychiatry respondents were satisfied with their quality of life (regional rates: East China, 18.6%; Central China, 14.7%; West China, 13.2%; Northeast China, 29.3%, respectively, *p* < 0.05), and 29.2% were satisfied with their health status (regional rates: East China, 28.1%; Central China, 32.1%; West China, 26.3%; Northeast China, 37.8%, respectively, *p* > 0.05). No gender differences were found for either quality of life or health status satisfaction.

### Common Reasons for Dissatisfaction and Job Satisfaction and Its Correlates

The top three reasons for job dissatisfaction were low pay (50.1%), contentious doctor-patient relationships (17.6%) and high workload (10.8%). Other reported reasons included low social status (7.6%), lack of support from friends and family (6.7%), poor administrative/leadership support (2.1%), and other reasons (5.1%).

18.7% of the residents surveyed had the intention to quit their jobs. When asked if they could choose again, 37.7% answered they would not choose the same job while more than one quarter (26.5%) answered they would. Nearly one half (48.1%) would not want their children to be residents and 41.2% did not wish their children to be medical doctors in other specialties.

The overall MSQ in this sample was 73.8 ± 13.8, suggesting respondents were moderately satisfied with their job. There was no significant gender difference in level of satisfaction (male 74.7 ± 14.5 vs. female 73.4±13.4, *p* > 0.05). Post-graduate Year (PGY)-1 residents reported the highest level of job satisfaction (76.2 ± 13.3), followed by PGY-5 (73.6 ± 14.9), PGY-2 (73.6 ± 11.9), PGY-4 (72.5 ± 14.5), and PGY-3 (71.2 ± 14.3) residents (overall *p* < 0.001). Significant differences in scores were seen across regions (regional scores: West China, 70.6 ± 13.7; Central China, 72.5 ± 14.1; East China, 75.7 ± 13.9; Northeast China, 79.0 ± 13.8, respectively, *p* < 0.05).

A multilevel modeling analysis was used to examine associations between individual factors and total MSQ scores. The results of fixed effects are shown in Table 3: fewer years of work, shorter work hours per week, higher monthly pay, having medical liability insurance, feeling satisfied with the level of cooperation between doctors and nurses, the hospitals' prevention/management of medical disputes, and the social environment for healthcare workers, were all significantly associated with higher job satisfaction.

**Table 3:** The association between individual- and hospital-level characteristics applying multilevel linear regression (*N* = 1,060).

**Dependent variable: MSQ score**	**Model 1 (null model)**			**Model 2**			**Model 3**		
	**Coef**.	**SE**	***P***	**Coef**.	**SE**	***p***	**Coef**.	**SE**	***p***
Intercept	73.1	1.0	<0.001^***^	59.6	1.1	<0.001^***^	60.0	1.9	<0.001^***^
**COMPOSITIONAL EFFECT**									
Age (centralized)				−0.7	0.4	0.099	−0.7	0.4	0.111
Gender (ref. female)				1.0	0.6	0.110	1.0	0.6	0.115
Marital status (ref. unmarried or other)				1.0	0.8	0.210	1.0	0.8	0.206
Children (ref. none)				0.4	0.9	0.647	0.4	0.9	0.674
Educational attainment (ref. college/medical school)				0.4	0.8	0.633	0.3	0.8	0.671
Have administrative position (ref. none)				1.6	3.5	0.643	1.6	3.5	0.644
**GRADE(ref. PGY-1)**									
PGY-2				−0.8	0.9	0.393	−0.8	0.9	0.384
PGY-3				−2.88	1.0	0.003^**^	−2.8	0.9	0.003^**^
PGY-4				−2.6	1.2	0.032^*^	−2.6	1.2	0.031^*^
PGY-5				−2.0	1.2	0.080	−2.0	1.2	0.079
Work hours per week (centralized)				−0.8	0.3	0.014^*^	−0.8	0.3	0.013^*^
Standardized pay per month in previous year (centralized)				0.9	0.3	0.006^**^	0.9	0.3	0.006^**^
Gap between expected and actual standardized monthly pay (centralized)				−0.7	0.3	0.022^*^	−0.7	0.3	0.023^*^
Medical liability insurance (ref. none or unknown)				1.7	0.6	0.008^**^	1.7	0.6	0.007^**^
Encountered medical disputes and complaints in previous year (ref. none)				0.1	0.7	0.202	0.1	0.7	0.839
Satisfied with medical disputes prevention (ref. not)				8.9	0.7	<0.001^***^	8.9	0.7	<0.001^***^
Satisfied with doctor-nurse cooperation (ref. not)				9.0	0.8	<0.001^***^	8.9	0.8	<0.001^***^
Satisfied with social environment for healthcare workers (ref. not)				7.5	0.8	<0.001^***^	7.5	0.8	<0.001^***^
**CONTEXTUAL EFFECT**									
Doctor-bed ratio							−2.0	8.6	0.818
**RANDOM PARAMETERS**									
Hospital level variance/standard error / *p*-value	26.5	8.0	0.001^***^	6.1	2.3	0.009	4.0	5.2	0.438
Individual level variance/standard error	162.6	7.2		88.7	3.9		88.7	3.9	
Intra-class correlation: ICC(%)	14.0			6.5			4.3		
−2 Log likelihood	8458.1			7759.9			7753.5		
Proportional changes in hospital level variance: PCV (%,compared to null model)				76.8					
Proportional changes in hospital level variance: PCV (%,compared to Model 2)							34.8		

*Coef., coefficient; SE, standard error; ref., reference*.

* p <0.05,

** p <0.01,

*** *p <0.001*.

## Discussion

This is the first nationwide survey focusing on the socio-demographic features, job satisfaction, and other related factors pertaining to psychiatry residents in China. We focused on their perceptions of the psychiatric profession, their reasons for dissatisfaction, and their quality of life. We found that the new generation of psychiatric trainees were predominantly female and they were well-educated, with more than one-third having a post-graduate degree in addition to their medical degree.

Overall, they were moderately satisfied and the common reasons for dissatisfaction were low pay, contentious patient-doctor relationships, and high workload. Nearly a fifth of participants was intentioned to quit their residency and less than a fifth were satisfied with their quality of life. Their salaries were lower than many professionals with comparable educational levels and medical disputes were common and demoralizing. Significant regional differences were observed in income levels, working hours and satisfaction levels, with those in West China being the worst.

Before we discuss our findings in detail, a few strengths of this survey need to be mentioned. The survey covered nearly all provinces and autonomous regions (29/31) in mainland China and the participation and completion rates were both very high. The survey was conducted online to ensure anonymity and reliability. All data were collected within a short time period (December 18th to 29th, 2017) and this ensured that the data were comparable cross-sectionally.

On the other hand, a few limitations of this study also need to be acknowledged. First, self-report studies have some inherent limitations, such as underreporting or exaggeration on some items or even misreporting, especially on sensitive items such as income. Second, because there are many local dialects in China, some survey questions might have been interpreted in different ways by different participants. For example, the Chinese term for medical disputes (“*Yiliao Jiufen*”) is very broad and inclusive leading different people to have different interpretations of the same phrase. Third, some important components of residency training were not included in the survey, such as call schedules, didactics, and supervision. Fourth, this study did not collect data on depressive symptoms and burnout. This is important as depression is common among medical students in Asia and the prevalence was 11.0% (20), and medical students who suffer from depression may choose psychiatry to better understand their illness (21). Furthermore, work-related disability and productivity loss associated with depression are critical determinants of career satisfaction (22). Finally, we were unable to collect any data from those who declined to participate so we cannot rule out the existence of selection bias.

### Basic Characteristics of Psychiatry Residents in China

In our sample, 34.3% psychiatry residents had a master's or doctoral degree in addition to their medical degree. This is in stark contrast to earlier reports such as one in 2013 which showed that 43.2% of psychiatric practitioners in China did not even have a medical degree (1). This was due to an historical shortage within the psychiatric workforce, resulting in some psychiatric practitioners only having an associate degree, especially those who joined the workforce before the 1990s. They often hold the lowest professional title and usually work under a supervising psychiatrist, similar to that of a nurse practitioner or a physician assistant in the U.S. This has improved significantly in the past 2 decades. Our findings show clear progress in terms of the educational background of the future psychiatric workforce.

Overall, the medical education system in China is similar to that in the United Kingdom and many other European countries. Medical college education starts immediately after high school and students receive a bachelor's degree in medicine. Medical school graduates then need to complete residency training before they can practice. Psychiatry residency lasts 5 years.

In 2013, among licensed physicians working in mental health facilities, 41.9% were female (1). In our survey, 70.0% of psychiatry residents were female. If this trend continues, the profile of the psychiatric workforce in China will change but the effects of this are unclear. Working an average of 47.8 h/week, psychiatry residents in China are exceeding the 40 h/week maximum set forth by Chinese Labor Laws (23). Of note, the reported working hours did not include the time spent studying for exams, research and other related issues. Surprisingly, the number of working hours was not one of the top contributors to job dissatisfaction suggesting that the work itself, rather than the number of hours, is the bigger problem.

### The Pay Level of Psychiatry Residents in China

Overall the monthly stipend of psychiatry residents in China was a median of RMB 6,961 (USD 1,050 or EUR 904.2). This is a relatively low amount given the cost of living in China. As is the case in the US, the stipends increase every year, however, the increases in China are small resulting in a median stipend of RBM 10,120 (USD 1,600 or EUR 1314.6) for the PGY-5 residents in our study. In this survey, low pay was the top reason for dissatisfaction (50.1%). Unfortunately, many physicians working in Chinese hospitals have lost their enthusiasm for continuing their career because of insufficient salaries, along with other factors (24).

The gender gap in expected pay is an interesting finding. While the actual pay was comparable between male and female residents, female residents' expected pay was significantly lower than that of their male colleagues. Some possible explanations are that female psychiatry residents have internalized the gender inequality embedded within Chinese culture (25, 26) and that society does not value female residents' professional efforts to the same extent that their male colleagues value their own efforts (27).

### Satisfaction, Dissatisfaction, and Intention to Quit

We found the level of job satisfaction among psychiatry residents in China as a whole was moderate. The most common reasons for residents' dissatisfaction were focused on low pay, contentious doctor-patient relationships, and high workload. In previous studies, psychiatry residents from other countries have identified items most likely to contribute to their overall satisfaction with their residency program. For example, based on their survey of 100 residents in Israel, Ellencweig et al. (28) found the top three reasons for satisfaction were quality of supervision, respect of faculty for residents, and program graduates' job satisfaction. Perhaps if training directors and other stakeholders in China focused on these issues in addition to trying to resolve the ones contributing to dissatisfaction, psychiatry residents in China would feel more satisfied. The high percentage of psychiatry residents who would not choose the same career is in sharp contrast to a recent report from the United States (29), which found 78% (the highest among all specialties) of young psychiatrists would choose psychiatry as their career and 82% would choose medicine if they had to do it all over again. Nearly a fifth of our residents reported an intention to quit residency. One study (30) of mental health professionals found that low job satisfaction directly predicted turnover intention and turnover plans. Another study (31) of physicians in the Hebei province in China found that turnover intention was significantly and negatively correlated with job-satisfaction levels. Clearly, more attention needs to be given to Chinese residents' job satisfaction if we are to reduce their desire to quit residency.

Less than a fifth (17.0%) of our sample was satisfied with their quality of life which is in sharp contrast with reports of psychiatry residents training in other countries (32). This low number is worrisome and educators and government officials should study this issue more closely.

### The High Rates of Medical Disputes and Low Liability Insurance Coverage

It is not surprising that nearly a third of psychiatry residents have experienced a medical dispute and 77.3% of psychiatry residents were dissatisfied with the social environment for healthcare workers; at the same time, 65.0% did not believe that they received their due respect from the general public. Over the past two decades, the social atmosphere and working environment for medical doctors has significantly deteriorated in China (33). Patients' mistrust of doctors is increasingly prevalent (34) and some argue that the doctor-patient relationship has reached an unprecedented level of tension in today's China (35). Although lack of funding and resources is believed to be the leading cause, another factor often mentioned is the media's negative and sensational coverage of hospitals and health professionals (36). Violence against doctors has been reported repeatedly (37–39) and the public image of psychiatry in China has suffered (40).

Medical disputes are often multifactorial and currently in China, the unique healthcare system (transitioning from the state-run system to a market oriented system) and limited resources are two often-mentioned factors contributing to high rates of medical disputes (41). In China, most disputes and complaints are reviewed and managed by hospital administrators. In our sample, only slightly more than half (52.7%) were satisfied with how their hospitals handled the disputes.

Significant regional differences were also observed. Medical liability insurance (malpractice insurance), which is often universal and mandatory in developed countries, is a relatively new development in China and it may work as a useful tool to cope with medical disputes (42). However, the development has been very slow and rigid (43). In this survey, only slightly more than half of participants (59.4%) had medical liability insurance coverage with a significant regional difference (West China was the lowest, 53.3%). This is an area that clearly needs improvement and governmental intervention.

### Regional Differences Among Psychiatry Residents

Regional differences were found among several of the variables studied. For example, residents in West China worked significantly longer hours, while those in Northeast China reported the lowest incomes, and those in Central China reported the most disputes. Factors that might have contributed to those differences include regional differences in culture (and unbalanced socioeconomic developments) occurring in some but not all regions. China is vast and unevenly developed. Different regions often have different resources and budgets for healthcare (44). Furthermore, the 32 participating hospitals varied in size, staffing, administrative style, and resources. All of these factors may also have contributed to the regional differences.

In summary, in this very first national survey of psychiatry residents training in China, we have findings that are encouraging and others that are more worrisome. On the one hand, the current psychiatry residents are well-educated and increasingly female, which, respectively, may help the reputation and diversity of the future workforce (45). On the other hand, we have discovered that these residents are only moderately satisfied in their job, many are considering quitting, and only a few are satisfied with their quality of life. Significant regional differences in income levels and working hours exist, with those in West China faring the worst. These findings call for greater support from the government, educators, mental health advocates, and hospital management to find solutions to improve residents' job satisfaction and wellness. These findings also point to areas where such improvement would be welcome: for example, residents' salary, the doctor-patient relationship, and access to medical liability insurance for all psychiatry residents. Without addressing these areas, the future of the psychiatric work force in China remains in jeopardy.

## Ethics Statement

This study was carried out in accordance with the recommendations of clinical research guidelines, the Ethical Committee of the School of Public Health, Peking Union Medical College with written informed consent from all subjects. All subjects gave written informed consent in accordance with the Declaration of Helsinki. The protocol was approved by the the Ethical Committee of the School of Public Health, Peking Union Medical College.

## Author Contributions

FJ, HZ, YT and YL were responsible for study design. FJ, HZ, LH, TL, SW, and HL contributed to data collection, analysis and interpretation. FJ, HZ, JR, YT, and YL drafted and revised the manuscript. All authors had full access to all the data in this study and approved the Abstract.

### Conflict of Interest Statement

The authors declare that the research was conducted in the absence of any commercial or financial relationships that could be construed as a potential conflict of interest.

## References

[1] Liu C Chen L Xie B Yan J Jin T Wu Z. Number and characteristics of medical professionals working in Chinese mental health facilities. Shanghai Arch Psychiatr. (2013) 25:277–85. 10.3969/2Fj.issn.1002-0829.2013.05.003 24991167 PMC4054572

[2] Wong VT Recruitment and training of psychiatrists in Hong Kong: what puts medical students off psychiatry–an international experience. Int Rev Psychiatr. (2013) 25:481–5. 10.3109/09540261.2013.816656 24032505

[3] Rios A Sanchez Gascon F Martinez Lage JF Guerrero M. Influence of residency training on personal stress and impairment in family life: analysis of related factors. Med Princ Pract. (2006) 15:276–80. 10.1159/000092990 16763394

[4] Ishak WW Lederer S Mandili C Nikravesh R Seligman L Vasa M. Burnout during residency training: a literature review. J Grad Med Educ. (2009) 1:236–42. 10.4300/JGME-D-09-00054.1 21975985 PMC2931238

[5] Abdulghani HM Al-Harbi MM Irshad M. Stress and its association with working efficiency of junior doctors during three postgraduate residency training programs. Neuropsychiatr Dis Treat. (2015) 11:3023–9. 10.2147/NDT.S92408 26677329 PMC4677768

[6] Ferrari S Cuoghi G Mattei G Carra E Volpe U Jovanovic N. Young and burnt? Italian contribution to the international BurnOut Syndrome Study (BOSS) among residents in psychiatry. Med Lav. (2015) 106:172–85. 25951864

[7] Scanlan JN Still M. Job satisfaction, burnout and turnover intention in occupational therapists working in mental health. Aust Occupa Ther J. (2013) 310–8. 10.1111/1440-1630.12067 24089982

[8] Huang L Hu S Wang H Cui HS Jin LJ Study on the relation of job burnout and turnover intention in the resident physician of general standardized training program. Shanghai Med Pharmaceut J. (2017) 38:7–11.

[9] Yin LX Zeng BW Kang L Correlation between stressor and job burnout of resident doctors. Chin J Med Manage Sci. (2014) 4:55–8.

[10] Jiang F Hu L Rakofsky J Liu T Wu S Zhao P. Sociodemographic characteristics and job satisfaction of psychiatrists in china: results from the first nationwide survey. Psychiatr Serv. (2018) 69:1245–51. 10.1176/appi.ps.201800197 30301449

[11] National Burearu Of Statistics of China The Method of Dividing East and West Central and Northeast China. (2011). Available online at: www.stats.gov.cn (Accessed March 12, 2018).

[12] Weiss DJ Manual for the Minnesota Satisfaction Questionnaire. Minneapolis, MN: Work Adjustment Project, Industrial Relations Center, University of Minnesota (1967).

[13] Liu ZY Wei WH Wang L Cui HZ Li YY Zhang FL The Relationship among job satisfaction, work engagement and organizational citizenship behavior of nurses. Chinese J Behav Med Brain Sci. (2017) 26:747–50.

[14] Wang J Zhao Q Yuan L Pan ZG Survey on work satisfaction among general practitioners in Shanghai. Chinese J General Pract. (2017) 16:921–5.

[15] Baessler F Riese F Pinto DCM de Picker L Kazakova O Kanellopoulos A. Becoming a psychiatrist in Europe: the title is recognized across the European Union, but what are the differences in training, salary and working hours? World Psychiatr. (2015) 14:372–3. 10.1002/wps.20259 26407800 PMC4592667

[16] National Bureau of Statistics of China 2016 Average Income Level in Each City. (2017). Available online at: www.stats.gov.cn (Accessed March 12, 2018).

[17] Rossi A Cetrano G Pertile R Rabbi L Donisi V Grigoletti L. Burnout, compassion fatigue, and compassion satisfaction among staff in community-based mental health services. Psychiatry Res. (2012) 200:933–8. 10.1016/j.psychres.2012.07.029 22951335

[18] Harvey Goldstein Mutilevel Statistical Models. 4th ed. Chichester: Wiley (2011).

[19] Pastor DA Lazowski RA. On the multilevel nature of meta-analysis: a tutorial, comparison of software programs, and discussion of analytic choices. Multivar Behav Res. (2018) 53:74–89. 10.1080/00273171.2017.1365684 28952787

[20] Cuttilan AN Sayampanathan AA Ho RC. Mental health issues amongst medical students in Asia: a systematic review [2000–2015]. Ann Transl Med. (2016) 4:72. 10.3978/2Fj.issn.2305-5839.2016.02.07 27004219 PMC4779785

[21] Flajsman AM Degmecic D Pranjkovic T Rogulja S Bosnjak D Kuzman MR. Medical education changes students' attitudes on psychiatry: survey among medical students in Croatia. Psychiatr Danub. (2017) 29(Suppl 4):859–65. 29278637

[22] Lee Y Rosenblat JD Lee J Carmona NE Subramaniapillai M Shekotikhina M. Efficacy of antidepressants on measures of workplace functioning in major depressive disorder: a systematic review. J Affect Disord. (2018) 227:406–15. 10.1016/j.jad.2017.11.003 29154157

[23] People's Congress of China The Labor Law of People's Congress of China. (1994). Available online at: www.jingbian.gov.cn (Accessed March 12, 2018).

[24] Zeng J Zeng XX Tu Q. A gloomy future for medical students in China. Lancet. (2013) 382:1878. 10.1016/S0140-6736(13)62624-0 24315177

[25] Zhang J Han J Liu PW Trends in the gender earnings differential in Urban China, 1988–2004. ILR Rev. (2008) 61:224–43. 10.1177/001979390806100205

[26] Ning GJ The gender gap in China's wages and its decomposition. In: World Economic Papers. (2011).

[27] He G Wu X. Marketization, occupational segregation, and gender earnings inequality in urban China. Soc Sci Res. (2017) 65:96–111. 10.1016/j.ssresearch.2016.12.001 28599783

[28] Ellencweig N Weizman A Fischel T. Factors determining satisfaction in psychiatry training in Israel. Acad Psychiatr. (2009) 33:169–73. 10.1176/appi.ap.33.2.169 19398637

[29] Peckham C Medscape Young Physician Compensation Report 2017. (2017). Available online at: https://www.medscape.com/slideshow/young-physician-compensation-2017-6008749 (Accessed May 21, 2018).

[30] Yanchus NJ Periard D Osatuke K. Further examination of predictors of turnover intention among mental health professionals. J Psychiatr Ment Health Nurs. (2017) 24:41–56. 10.1111/jpm.12354 27928857

[31] Zhang Y Feng X. The relationship between job satisfaction, burnout, and turnover intention among physicians from urban state-owned medical institutions in Hubei, China: a cross-sectional study. BMC Health Serv Res. (2011) 11:235. 10.1186/1472-6963-11-235 21943042 PMC3197494

[32] Kovach JG Combs CJ Singh H Dubin WR. Psychiatry resident quality of life. Acad Psychiatr. (2016) 40:76–80. 10.1007/s40596-015-0387-9 26122355

[33] Wu LX Qi L Li Y. Challenges faced by young Chinese doctors. Lancet. (2016) 387:1617. 10.1016/S0140-6736(16)30202-1 27116071

[34] Nie JB Cheng Y Zou X Gong N Tucker JD Wong B. The vicious circle of patient-physician mistrust in China: health professionals' perspectives, institutional conflict of interest, and building trust through medical professionalism. Dev World Bioeth. (2018) 18:26–36. 10.1111/dewb.12170 28922547

[35] Jing W Otten H Sullivan L Lovell-Simons L Granek-Catarivas M Fritzsche K. Improving the doctor-patient relationship in China: the role of balint groups. Int J Psychiatry Med. (2013) 46:417–27. 10.2190/PM.46.4.g 24922991

[36] Yang Z Liu Y Fan D. Workplace violence against health care workers in the United States. N Engl J Med. (2016) 375:e14. 10.1056/NEJMc1606816 27532858

[37] Wang XQ Wang XT Zheng JJ. How to end violence against doctors in China. Lancet. (2012) 380:647–8. 10.1016/S0140-6736(12)61367-1 22901881

[38] Tucker JD Cheng Y Wong B Gong N Nie JB Zhu W. Patient-physician mistrust and violence against physicians in Guangdong Province, China: a qualitative study. BMJ Open. (2015) 5:e008221. 10.1136/bmjopen-2015-008221 26443652 PMC4606416

[39] Sun P Zhang X Sun Y Ma H Jiao M Xing K. Workplace violence against health care workers in north Chinese hospitals: a cross-sectional survey. Int J Environ Res Public Health. (2017) 14:E96. 10.3390/ijerph14010096 28106851 PMC5295346

[40] Liu XL Wu CD Lei MQ Comparison of medical disputes causes between mental hospital and general hospital. China Med Herald. (2014) 11:147–50.

[41] Niu SZ Yan JF Niu MY Analysis of the current situation of medical disputes and countermeasures. Legal Syst Soc. (2016) 10:185–6.

[42] Abbott RL Weber P Kelley B. Medical professional liability insurance and its relation to medical error and healthcare risk management for the practicing physician. Am J Ophthalmol. (2005) 140:1106–11. 10.1016/j.ajo.2005.07.020 16376659

[43] Sun XQ Peng H Guo XJ Chen Z Qing MW Analysis and reflection on the effect of medical liability insurance in the hospital for ten years. Chines Hospital Manage. (2016) 36:88–9.

[44] Dong EH Li GH Cai YY Wang T A review on regional difference in healthcare resource allocation. Chinese Health Resour. (2016) 19:390–3.

[45] Liu J Yan F Ma X Guo HL Tang YL Rakofsky JJ. Perceptions of public attitudes towards persons with mental illness in Beijing, China: results from a representative survey. Soc Psychiatr Psychiatr Epidemiol. (2016) 51:443–53. 10.1007/s00127-015-1125-z 26510417

